# Hypertryptasemia and Mast Cell-Related Disorders in Severe Osteoporotic Patients

**DOI:** 10.1155/2020/5785378

**Published:** 2020-10-20

**Authors:** Giulia Carosi, Gregorio Guabello, Matteo Longhi, Federica Grifoni, Elena Passeri, Sabrina Corbetta

**Affiliations:** ^1^Endocrinology Unit, IRCCS Fondazione Ca' Granda Ospedale Maggiore Policlinico, Milan, Italy; ^2^Department of Experimental Medicine, Sapienza University of Rome, Rome, Italy; ^3^Rheumatology Unit, IRCCS Istituto Ortopedico Galeazzi, Milan, Italy; ^4^Hematology and Transplantation Unit, IRCCS Fondazione Ca' Granda Ospedale Maggiore Policlinico, Milan, Italy; ^5^Endocrinology and Diabetology Service, IRCCS Istituto Ortopedico Galeazzi, Milan, Italy; ^6^Department of Biomedical, Surgical and Dental Sciences, University of Milan, Milan, Italy

## Abstract

**Purpose:**

Systemic mastocytosis (SM) is characterized by a clonal proliferation of neoplastic mast cells (MCs) in one or more extracutaneous organs including the bone marrow (BM). SM is often associated with osteoporosis (OP) and fractures. Hypertryptasemia usually occurs in SM. We investigated the prevalence of hypertryptasemia in a series of severe osteoporotic patients, the performance of the tryptase test in diagnosing SM in these patients, and their bone features.

**Methods:**

The medical records of 232 patients (168 females and 64 males) with a diagnosis of OP (50.4% with fractures) and a serum tryptase assessment were reviewed. BM assessment was performed in a subset of hypertryptasemic patients; clinical, biochemical, and radiographic data were collected.

**Results:**

Hypertryptasemia was detected in 33 patients. BM assessment (*n* = 16) was normal in 8 hypertryptasemic patients, while BM criteria for the diagnosis of SM were met in 3 patients, MC alterations were detected in 4 patients, and one patient presented a polycythemia vera. Serum tryptase levels were higher than 11.4 ng/ml in all patients with BM alterations. The best cut-off of tryptase level related to BM alterations was 17.9 ng/ml, with a sensibility and sensitivity of 75% (AUC = 0.797 and *P* = 0.015 by ROC analysis). All osteoporotic patients with hypertryptasemia experienced at least one vertebral fracture associated with a severe reduction of the lumbar bone mineral density.

**Conclusions:**

The prevalence of MC-related disorders in severe OP was 3.0%, accounting for the 7.4% of the secondary causes of OP. MC-related disorders may be involved in bone fragility and assessment of serum tryptase is useful to detect MC-related disorders.

## 1. Introduction

Osteoporosis (OP) is a systemic skeletal disorder characterized by decreased bone mass and impairment of bone quality, which is associated with increased risk of fragility fractures. Several diseases and drugs are known to contribute to OP development. International guidelines recommend screening for secondary cause of OP in order to assess the fracture probability [1–3]. Diagnostic workup for the secondary causes of OP requires extensive biochemical evaluation. In this context, the assessment of serum tryptase, a mastocyte cell-specific serine protease, could be considered in order to rule out systemic mastocytosis, a rare hematological disorder generally associated with hypertryptasemia [4]. Mastocytosis comprises a heterogeneous group of disorders characterized by expansion and accumulation of neoplastic mast cells (MCs) in one or more organ systems. In patients with systemic mastocytosis (SM), neoplastic MCs form focal and/or diffuse infiltrates in various internal organs, including the bone marrow (BM), spleen, liver, and gastrointestinal tract [5]. Epidemiological data are sparse in the general population, reporting a prevalence of 0.5-1.0 per 10,000, which is probably underestimated [5, 6]. Two main variants of the disorder are described: cutaneous mastocytosis, generally observed in the childhood, and systemic mastocytosis (SM), an adult disease. While the cutaneous mastocytosis spontaneously regresses in most cases, SM is a persistent disease, and it can evolve in more aggressive disorders, namely, aggressive SM or mast cell leukemia. In most adult patients, a gain-of-function somatic mutation in the *KIT* gene, encoding for the stem cell growth factor receptor, is detected, the D816V mutation being the most frequent. Symptoms and signs are related to MC proinflammatory and vasoactive mediators' release (e.g., flushing, urticaria, and anaphylaxis) and to MC infiltration (e.g., spleen enlargement) [7]. Diagnostic criteria have recently been updated on current WHO classification [5, 8, 9]; they include a biochemical finding, namely, serum tryptase levels higher than 20.0 ng/ml and histopathological, morphological, cytofluorimetric, and molecular criteria resulted from a bone marrow evaluation with bone marrow biopsy (BMB) and bone marrow aspirate (BMA). Three minor criteria (persistent tryptase levels > 20.0 ng/ml, CD25 with or without CD2 aberrant expressions, abnormal mast cells morphology, and detection of the D816V *KIT* mutation) or one minor associated with the major criteria (i.e., multifocal dense mast cell aggregates) have to be met.

Some studies described a high prevalence of bone fragility in patients affected with SM, up to 33% [10–13], suggesting SM as a cause of secondary OP. Besides, no consistent data on SM prevalence in osteoporotic patients are available. The present study is aimed at assessing the prevalence of hypertryptasemia and SM in a series of patients with unexplained OP and/or skeletal fragility, who were evaluated looking for secondary OP.

## 2. Subjects and Methods

The study was carried out at the IRCCS Istituto Ortopedico Galeazzi in Milan, where osteoporotic patients firstly referred to the Rheumatology and Endocrinology Units were enrolled. The diagnostic workup for SM was performed at the Hematology and Transplantation Unit, IRCCS Fondazione Ca' Granda Ospedale Maggiore Policlinico, Milan. We retrospectively analyzed the records of a series of referred patients from May 2014 to July 2018 presenting two main features: a diagnosis of OP and a serum tryptase assessment in their medical records. OP was assessed using bone mineral density (BMD) measurement at lumbar spine and femur by dual-energy X-ray absorptiometry (DXA) technique. In postmenopausal women and men aged 50 years and older, a diagnosis of OP was made in presence of a *T* − score < −2.5 at any site according to WHO criteria [14]. In premenopausal women and men less than 50 years, *Z*-scores were used, considering *Z*-score values of −2.0 or lower as reduced BMD for age. All patients with fragility fractures were considered as affected with severe OP, regardless of BMD values [1]. Data about secondary OP causes as well as comorbidities and concomitant treatments reported in patients' medical history were collected. All patients were screened for secondary causes of OP by an extensive biochemical workup, and data were collected. Data about BMD and bone fractures were also recorded in an electronic database.

Serum tryptase was routinely assessed in patients with prevalent vertebral fractures and/or severe bone demineralization at vertebral site (*T* − score < −3.0 by DXA) in line with previous reports (reviewed in [15]) describing high prevalence of vertebral bone impairment in patients with SM.

All procedures performed in studies involving human participants were in accordance with the ethical standards of the institutional committee and with the 1964 Helsinki declaration and its later amendments or comparable ethical standards. The study follows the STROBE guidelines for observational studies.

### 2.1. Biochemical Screening for Secondary OP Detection

Patients were assessed for the occurrence of hypercalciuria (24-hour urine collection calcium), hypophosphatemia (serum phosphate and 24-hour urine collection phosphate), thyroid dysfunction (serum TSH levels), male hypogonadism (calculated free testosterone, LH, and FSH), primary and secondary hyperparathyroidism (serum calcium and PTH), hypophosphatasia (serum total ALP), hypercortisolism (low-dose dexamethasone suppression test), multiple myeloma and monoclonal gammopathies (MGUS) (serum protein electrophoresis and urinary immunofixation test), hematologic disorders (hemocromocytometry), rheumatoid arthritis and connectivities (rheumatoid factor test, ANA, ENA, and ESR), chronic liver diseases (AST, ALT, and *γ*GT), and chronic kidney diseases (serum creatinine and estimated glomerular filtration rate).

### 2.2. Definition of Hypertryptasemia

Serum tryptase assessment was performed in different laboratories using the same assay method (ImmunoCAP Tryptase, Phadia Laboratory Systems, Thermo Fisher Scientific Inc., Uppsala, Sweden). The upper limit of the normal reference intervals varied among the laboratories ranging from 5.0 to 11.4 ng/ml [16].

Serum tryptase levels higher than 11.4 ng/ml and 20.0 ng/ml were considered suspicious and strongly suspicious for SM, respectively, according to the current most widely accepted ranges [4, 7].

### 2.3. Diagnosis of Systemic Mastocytosis (SM)

Diagnosis of SM was carried out according to current WHO criteria based on bone marrow assessment and biochemical findings [4, 7, 17], including immunohistochemistry staining for tryptase, serum tryptase levels, analysis of CD2/CD25 expression on mast cells by flow or immunohistochemistry, and genotyping of *cKIT* (D816V) mutation. *cKIT* mutation was evaluated by reverse transcriptase polymerase chain reaction (RT-PCR) restriction fragment length polymorphism (RFLP) analysis or direct Sanger sequencing.

Based on the associated clinical features, SM was further classified as reported in the legend of Table 1 [17].

### 2.4. Statistical Analysis

Data are presented as mean ± standard deviation (SD), and percentages where not otherwise stated. *T*-test was used to compare normally distributed continuous variables. Normality was tested by D'Agostino-Pearson test. Correlation analyses between continuous and categorical data were performed using Spearman's correlation. Receiver operator characteristic (ROC) analysis was performed to assess threshold value of serum tryptase that detects patients with bone marrow alterations. The analyses were performed with Prism 6.0.

## 3. Results

### 3.1. Prevalence of Hypertryptasemia in Severe Osteoporotic Patients

The medical records of 232 outpatients (168 females and 64 males) evaluated for bone fragility from May 2014 to July 2018 to the third-level center IRCCS Istituto Ortopedico Galeazzi in Milan presenting a diagnosis of OP and a serum tryptase assessment were reviewed. The mean age at the first evaluation was 64.0 ± 11.0 and 63.0 ± 13.0 years for females and males, respectively. Osteoporotic fractures occurred in 117 out of 232 patients (50.4%). In the 232 evaluated patients, mean tryptase level (±SD) was 7.1 ± 6.0 ng/ml, with any significant difference between males (7.4 ± 5.8 ng/ml) and females (7.0 ± 6.1 ng/ml; *P* = 0.735). In the whole series, serum tryptase levels did not correlate with *T*-scores or *Z*-scores (*P* = 0.33 and 0.27 for lumbar spine *T*-scores and *Z*-scores, respectively; *P* = 0.25 and 0.27 for femoral neck *T*-scores and *Z*-scores, respectively; *P* = 0.59 and 0.52 for total hip *T*-scores and *Z*-scores, respectively; analyzed by Spearman's correlation).

According to local laboratories' reference ranges, hypertryptasemia was detected in 33 patients, indicating a prevalence of 14.2%. None of the hypertryptasemic patients was affected with severe chronic kidney disease (defined as stage 3 or higher stage CKD) or with known hematological disorders determining tryptase elevation. Hypertryptasemic osteoporotic patients did not report symptoms related to the tryptase release; moreover, hepatomegaly, splenomegaly, and/or lymphadenopathy were not recorded on physical examination, and cytopenia was absent in all patients.

### 3.2. BM Findings in Osteoporotic Patients with Hypertryptasemia

**Figure 1:**
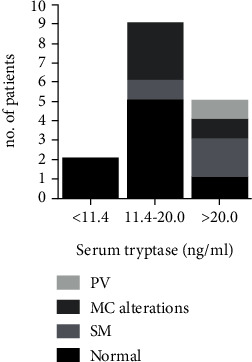
Bone marrow assessment results related to serum tryptase levels. MC: mastocyte cells; SM: systemic mastocytosis; PV: polycythemia vera.

BM data were available from 16 out the 33 identified hypertryptasemic osteoporotic patients, and the remaining 17 refused the procedure (serum tryptase levels 19.2 ± 0.1 vs. 9.7 ± 7.2 ng/ml, respectively; *P* = 0.003). BM assessment was performed in 2 out of 17 patients with serum tryptase levels lower than or equal to 11.4 ng/ml, in 9 out of 9 patients with serum tryptase levels within the interval 11.5-20.0 ng/ml, and in 5 out of 7 patients with tryptase levels higher than 20.0 ng/ml (Figure 1).

BM assessment revealed normal bone marrow parameters in 8 out of 16 hypertryptasemic patients, while in the remaining 8 bone marrow alterations could be detected. Specifically, criteria for the diagnosis of SM [5, 8, 9] could be unequivocally defined in 3 patients. In additional 4 patients, MC alterations in BM, not fully matching WHO criteria for SM diagnosis, were detected (Table 1).

MC alterations were consistent with abnormal mast cell morphology in 1 patient and with CD25 aberrant expression in 3. Lastly, in 1 patient, BM findings diagnosed the previously unrecognized myeloproliferative disorder polycythemia vera harboring the *JAK2* gene mutation. Considering patients with both SM and MC alterations, the prevalence of mast cell disorders in severe osteoporotic patients was 3.0%.

All the 8 patients harboring MC-related BM abnormalities showed a serum tryptase level higher than 11.4 ng/ml, which were below the threshold of 20.0 ng/ml in 4 patients and higher than 20.0 ng/ml in the remaining 4 patients (Table 1, Figure 1).

The ROC analyses indicated 17.9 ng/ml as the best cut-off of tryptase level related to BM alterations, with a sensibility and sensitivity of 75% (AUC = 0.797, *P* = 0.015). Accordingly, considering the threshold of 20.0 ng/ml, which is generally considered as highly suggestive of SM, tryptase assessment showed a positive predictive value of 0.80, while the negative predictive value was 0.36 in detecting MC-related bone marrow alterations in the present series of osteoporotic patients.

### 3.3. Calcium and Bone Metabolism Parameters in Patients with SM and MC Alterations

None of the patients with diagnosis of MS and MC alterations showed abnormal calcium and bone metabolism parameters; in particular, they were normocalcemic and normophosphatemic, with serum PTH levels in the normal low range and serum 25OHD levels above 30 ng/ml in 5 out of 7; total alkaline phosphatase levels were in the normal high range (Table 2).

Any significant difference could be detected in the mineral metabolism parameters between patients with and without BM alterations: mean serum phosphate levels were 3.5 ± 0.4 vs. 3.3 ± 0.5 mg/dl, *P* = 0.46; total ALP 101.4 ± 17.0 vs. 63.8 ± 16.0 U/L, *P* = 0.08; 25OHD 31.0 ± 7.0 vs. 31.4 ± 6.7 ng/ml, *P* = 0.94; PTH 32.6 ± 11.0 vs. 36.5 ± 5.8 pg/ml, *P* = 0.54. Indeed, mean serum calcium level was slightly higher in patients with BM alterations with respect to patients without BM alterations (9.6 ± 0.3 vs. 9.2 ± 0.2 mg/dl, *P* = 0.03), though both were within the normal range.

### 3.4. Bone Features in Patients with SM and MC Alterations

Skeletal involvement, with osteolytic lesions occurring in the skull, ribs, humeral bones, and pelvis, was detected in only one patient (no. 1) with SM diagnosis (Table 3).

**Figure 2:**
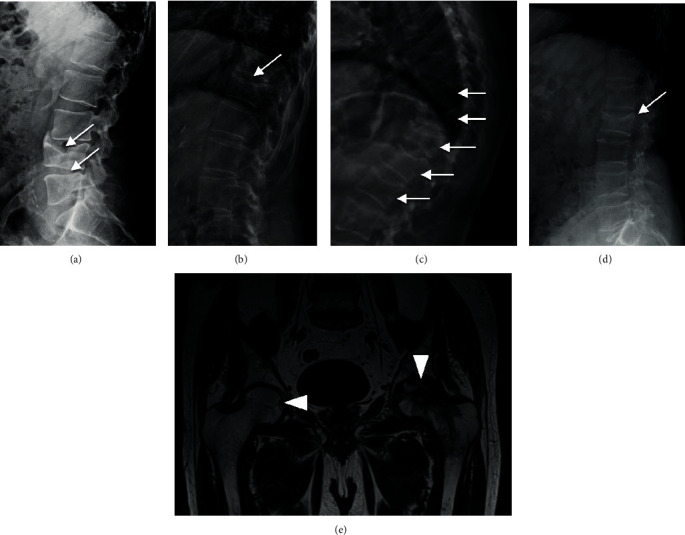
Radiological skeletal findings in patients with SM and MC alterations: (a) L4-L5 vertebral biconcave deformities (white arrows) in patient no. 2 with SM diagnosis; (b) T11 vertebral deformity (white arrow) in patient no. 3 with SM; (c) multiple vertebral biconcave deformities (L2-T10) (white arrows) in patient no. 7 with MC alterations; (d) L2 vertebral biconcave deformity (white arrow); (e) NMR image of the bilateral necrosis of femur heads in patient no. 6 with MC alterations; initial necrotic lesion of the right femoral head (white arrowhead on the left) and extensive necrosis of the left femoral head (white arrowhead on the right). SM: systemic mastocytosis; MC: mast cells; NMR: nuclear magnetic resonance.

All the patients with SM or MC alterations experienced at least one vertebral fracture, while any fracture at alternative sites was reported (Table 3). About half of the vertebral fractures were symptomatic and clinically evident, while the remaining was diagnosed by X-ray vertebral morphometry. In the present series, vertebral fractures were characterized by biconcave deformation, similarly to what reported by Kanitez et al. [18] (Figures 2(a)–2(d)). Indeed, additional bone alterations were detected in patients with MS or MC alterations: one patient (no. 6) experienced bilateral aseptic femur head necrosis 4 years before the occurrence of the vertebral fracture and the diagnosis of MC alteration (Figure 2(e)).

Spinal impairment occurred in 87.5% of patients with BM alterations, while it could be detected only in 37.5% of patients with normal BM result (*P* = 0.11). Patients with SM or MC alterations showed a more severe reduction of the lumbar BMDs with respect to the femur BMDs, with the exception of patient no. 5; indeed, she was affected with severe lumbar calcifying enthesopathy (Table 2). Comparing densitometric values of patients with BM alterations with all normotryptasemic patients, we observed that spinal *T*-scores resulted lower in patients with BM alterations (mean lumbar spine *T*-score, -3.8 vs. -2.8; *P* = 0.02), while the other densitometric parameters were similar.

Patients treated with antiresorptive drugs did not experience any incident fracture, and two patients showed increasing BMDs (Table 3); indeed, the median clinical follow-up was limited [12 months (6-84 months)]. Though any correlation could be detected between bone mineralization, in terms of *T*-scores, and the serum tryptase levels, the number of vertebral fractures showed a trend towards a positive correlation with the serum tryptase levels in osteoporotic patients with hypertryptasemia suggestive for SM (serum tryptase levels > 11.4 ng/ml; *r* = 0.239 and *P* = 0.054). Besides, a significant positive correlation between the number of vertebral fractures and the tryptase levels could be detected in patients with SM/MC alterations (*r* = 0.778and *P* = 0.01).

### 3.5. Prevalence of the SM and MC Alterations among the Secondary Causes of Osteoporosis

**Figure 3:**
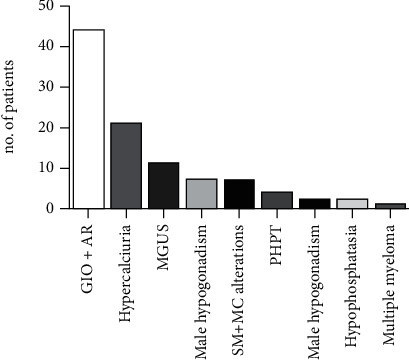
Causes of secondary osteoporosis in the analyzed patients' series. *GIO*: glucocorticoid-induced osteoporosis; AR: rheumatoid arthritis and connectivities; *hypercalciuria*: idiopathic hypercalciuria; *MGUS*: monoclonal gammopathy of undetermined significance; *PHPT*: primary hyperparathyroidism; *SM*: systemic mastocytosis; *MC*: mast cells.

In 95 (41%) out of the 232 patients with serum tryptase assessment, secondary OP was diagnosed. In 51 (22%), the cause of OP was evident only after the extensive biochemical and hormonal diagnostic workup for secondary OP (Figure 3). SM accounts for about 3.1% of the cases with secondary OP, while considering also the MC alterations, the prevalence raised to 7.4%.

## 4. Discussion

A growing number of diseases contribute to bone demineralization and/or to increasing the bone fracture risk. Osteoporosis and fragility fractures are often clinical features of systemic diseases, whose diagnosis is mandatory in the management of osteoporosis. Disorders of the bone marrow cells affect skeletal bone metabolism. Bone manifestations are one of the most frequent symptoms of SM, particularly in adults. Osteoporosis in SM has been attributed either to neoplastic infiltration or to the local release of mediators (histamine, heparin, tryptase, lipid mediators, and cytokines) [15]; bone fragility may be also related to osteolytic lesions. Patients may experience a wide spectrum of bone symptoms from poorly localized bone pain, diffuse osteopenia, or osteoporosis with fragility fractures, to diffuse osteosclerosis, or both focal osteolytic and osteosclerotic bone lesions [10, 12, 18]. The prevalence of osteoporosis in adult patients with SM is uncertain, though it has been estimated to occur in up to one-third of patients with indolent SM [15]. Nonetheless, osteoporosis may be the symptom of presentation of bone marrow SM [15, 19]. Hypertryptasemia represents the circulating biochemical marker suggestive for the diagnosis of SM, though cases of SM with normal tryptase levels have been reported [15]. It often reflects the mast cell burden, and tryptasemia persistently higher than 20.0 ng/ml is one of the minor criteria for the diagnosis of SM [8, 9].

By retrospectively investigating the prevalence of hypertryptasemia in severe osteoporotic patients, defined by the occurrence of fragility fractures and/or lumbar spine *T* − score < −3.0, in the setting of the extensive biochemical workup for the screening of secondary causes of osteoporosis, we report that hypertryptasemia occurred in about 14% of the patients, suggesting that including serum tryptase assay in the screening of osteoporotic patients for secondary causes will induce clinicians to familiarize with and to move on with further analysis.

Bone marrow is almost always involved in SM; therefore, BM histopathological evaluation is crucial to establish the diagnosis of SM, to assess tissue burden of MCs, and to rule out the presence of other hematological disorders. In the present series of hypertryptasemic osteoporotic patients, BM assessment diagnosed SM in about 19% of patients and identified MC alterations in further 25% of patients. Therefore, considering the whole series of osteoporotic patients with serum tryptase assessment, the prevalence of SM was 1.3%, and, including the MC alterations, it increased to 3.0%. These frequencies are similar to those previously reported in a series of iliac crest bone biopsies from osteoporotic patients (1.3%) [20] and in a series of diagnostic biopsies in fractured vertebrae (3.1%) [21].

Considering the threshold of 20.0 ng/ml, the diagnostic serum tryptase levels for SM [4, 7, 17], tryptase test showed high sensitivity but low specificity in osteoporotic patients, as 40% of patients with serum tryptase levels ranging 11.4-20.0 ng/ml showed MC alterations in BM, suggesting that in this set of patients, MC alterations and/or SM should be considered also when tryptase is included in the range 11.4-20.0 ng/ml. Accordingly, ROC analysis in the present series of hypertryptasemic osteoporotic patients identified 17.9 ng/ml as the best cut-off associated with MC alterations.

Moreover, hypertryptasemia may be associated with other hematologic diseases such as polycythemia vera also in osteoporotic patients. Increased tryptase levels may be consequent to a number of concomitant conditions: chronic urticaria, acute anaphylactic reactions, renal insufficiency, other hematological diseases, onchocercosis, and ischemic myocardial disease or in the presence of heterophile antibodies [22]. None of these conditions could be detected in the hypertryptasemic osteoporotic patients with bone marrow normal findings. Recently, a hereditary autosomal dominant form of hypertryptasemia, caused by increased germline copies of the *TPSAB1* gene encoding alpha-tryptase, has been recognized [22–25]. However, genetic analysis could not be performed in the present series of hypertryptasemic patients.

Considering the bone fragility, previous studies reported a typical spine involvement, regarding both fractures and BMD values [13, 15]. We actually observed a high prevalence of vertebral fractures in SM patients, mostly multiple, in agreement with previous studies [26–28], and the spine represented the unique site of fracture. The differences between spine and femoral BMDs are noticeable, with a consistent impairment in spine BMD values. Indeed, a previous study reported lower femur *T*-score, besides older age at diagnosis and elevated bone turnover markers, as risk factors for osteoporosis and fragility fractures in SM patients [26]. Moreover, vertebral fractures were characterized by biconcave deformities as reported in previous studies [12, 15, 29], but we found additional bone alterations in patients with SM and MC alterations, such as osteolytic lesions and bilateral necrosis of the femur heads.

At variance with previous reports, in the present study, we also consider the finding of MC alterations in BM, which do not match the major criteria for SM diagnosis. These patients, according to some authors, may have a “prediagnostic SM,” whose natural history needs to be defined [4].

Though the number of patients reported in the present series is limited, the prevalent vertebral BMD impairment, the occurrence of multiple vertebral fractures, and the biconcave deformities are similar to those observed in patients with diagnosis of SM. Noteworthy, the number of vertebral fractures positively correlated with the serum tryptase levels in osteoporotic patients with hypertryptasemia and MC alterations. Our data suggest that MS as well as MC alterations may contribute to bone fragility in addition to other osteoporotic-related disorders, such as male hypogonadism, diabetes mellitus, kidney stones, inflammatory bowel diseases, and pregnancy.

Finally, MC-related disorders emerged as a significant cause of secondary OP accounting for about 7% of cases and should be considered in patients with prevalent vertebral impairment, in terms of both BMD and fractures.

Admittedly, the study suffered from some limits: (1) it was a retrospective analysis; (2) it is likely that patients with evident causes of secondary OP did not receive a tryptase assessment, leading to an underestimated prevalence of the MC-related disorders in osteoporotic patients; (3) the study design did not allow to establish a pathogenic role of MC-related disorders in osteoporosis and bone fragility. Nonetheless, the present data may suggest further investigation about the role of tryptase and/or mast cell proliferation in the modulation of bone metabolism.

In conclusion, hypertryptasemia is a frequent finding in unexplained severe osteoporotic patients and bone marrow SM represents a cause of secondary OP in up to 1.3% of cases. We suggest of assessing serum tryptase levels in patients with bone fragility, particularly in the presence of spine BMD impairment and multiple vertebral fractures, even in the absence of classical signs and symptoms of MC activation usually reported in MS.

## Figures and Tables

**Table 1:** Bone marrow assessment and clinical findings in osteoporotic patients with hypertryptasemia.

Pts	Sex	Age years	Serum tryptase (ng/ml)	Diagnosis	Bone marrow histology	Major criterion^∗^	Minor criteria^∗^				Comorbidities
							1	2	3	4	
1	M	62	24.0	SM	Rare interstitial, perivascular, and paratrabecular MCs	-	-	+	+	+	Hypogonadism
2	M	41	26.0	SM	No MC infiltration, no MC granuloma	+	-	-	+	-	Active smoke, hypogonadism, alcoholism
3	F	65	19.2	SM	No MC infiltration, no MC granuloma	-	+	+	+	-	-
4	F	57	23.0	MCs alterations	No MC infiltration, no MC granuloma	-	+	-	-	-	-
5	M	70	16.3	MCs alterations	No MC infiltration, no MC granuloma	-	-	+	-	-	Hypogonadism, DM2, CKD, psoriatic arthropathy
6	M	73	15.4	MCs alterations	No MC infiltration, no MC granuloma	-	-	-	+	-	Adrenal incidentaloma, kidney stones, psoriasis
7	F	41	48.4	MCs alterations	Perivascular MC aggregates	-	-	-	+	+	IBD, pregnancy, lactation
8	M	79	21.0	PV	-	-	-	-	-	-	Previous prostate cancer, coronaropathy
9	M	62	12.7	Normal	-	-	-	-	-	-	Active smoke, MS
10	M	73	11.5	Normal	-	-	-	-	-	-	COPD
11	F	67	13.2	Normal	-	-	-	-	-	-	-
12	M	52	16.0	Normal	-	-	-	-	-	-	-
13	F	50	11.4	Normal	-	-	-	-	-	-	COPD, hypothyroidism
14	F	42	16.9	Normal	-	-	-	-	-	-	Previous anorexia nervosa
15	F	65	26	Normal	-	-	-	-	-	-	Iatrogenic thyrotoxicosis
16	M	79	18	Normal	-	-	-	-	-	-	Coronaropathy, MGUS

BM: bone marrow; SM: systemic mastocytosis; MCs: mast cells; PV: polycythemia vera; DM2: type 2 diabetes mellitus; CKD: chronic kidney disease; IBD: inflammatory bowel disease; MS: metabolic syndrome; COPD: chronic obstructive pulmonary disease. ^∗^The diagnosis of SM can be made when the major criterion and at least 1 minor criterion are present or when ≥3 minor criteria are present [8, 9]. Major criterion: multifocal dense infiltrates of MCs (≥15 MCs in aggregates) in BM biopsies and/or in sections of other extracutaneous organ(s). Minor criteria: (1) >25% of all MCs are atypical cells (type I or type II) on BM smears or are spindle shaped in MC infiltrates detected on sections of visceral organs. (2) KIT point mutation at codon 816 in the BM or another extracutaneous organ. (3) MCs in BM or blood or another extracutaneous organ exhibit CD2 and/or CD25. (4) Baseline serum tryptase level > 20 ng/ml (in case of an unrelated myeloid neoplasm, item “d” is not valid as an SM criterion).

**Table 2:** Mineral metabolic features in patients with SM and MC alterations.

Pts	Diagnosis	History of allergy/anaphylaxis	Ca	P	PTH	ALP	25OHD
			mg/dl	mg/dl	pg/ml	U/L	ng/ml
n.v.			8.4-10.4	2.8-5.0	10.0-65.0	40-120	>30
1	SM	Yes	9.6	3.8	26.0	116	41
2	SM	No	9.7	3.7	30.0	86	32
3	SM	No	9.6	3.5	33.0	110	22
4	MC alterations	No	9.8	3.5	23.0	90	31
5	MC alterations	No	9.8	2.8	21.0	117	23
6	MC alterations	No	9.2	3.3	49.5	76	38
7	MC alterations	No	9.2	3.9	45.5	115	30

Pts: patients; n.v.: normal values; Ca: serum total calcium; P: serum phosphate; PTH: serum parathyroid hormone; ALP: serum total alkaline phosphatase activity; 25OHD: serum 25-hydroxyvitamin D; SM: systemic mastocytosis; MC alterations: mast cell alterations.

**Table 3:** Bone mineral densities and vertebral fractures in patients with SM and MC alterations.

Patient	Diagnosis	LS *T*s	LS *Z*s	FN *T*s	FN *Z*s	TH *T*s	TH *Z*s	Vertebral fracture	Treatment	Treatment efficacy (follow-up)
1	SM	-4.30		-2.80		-1.80		T6, T8, T9, T11, T12, L1, L3	Alendronate 70 mg/week	No incident fractures (12 months)
2	SM		n.a.		-1.50		-1.00	T6, L4, L5	Zoledronate 5 mg/12 months	No incident fractures (12 months)
3	SM	-4.20		-2.30		-2.40		T12	Alendronate 70 mg/week	No incident fractures (24 months)
4	MC alterations	-3.40		-2.50		-2.30		T7, T8, T9, L2, L3, L4	Teriparatide 20 mcg/day+denosumab 60 mg/25 weeks	No incident fractures and significant increases in BMDs (24 + 12 months)
5	MC alterations	0.30^∗^		-1.60		-0.20		T11, T12	Treatment refused	Not available
6	MC alterations	-4.30		-2.80		-2.60		L2	Alendronate 70 mg/week	No incident fractures (12 months)
7	MC alterations		-3.90		-1.50		-1.30	T5, T6, T8, T10, T11, T12, L1, L2, L3, L5	Alendronate 70 mg/week	Not available (6 months)
8	PV	-2.8		-2.4		-1.7		No fracture detected	Risedronate 35 mg/week	Significant increases in BMDs (84 months)

^∗^Severe lumbar enthesophaties; SM: systemic mastocytosis; MC: mastocytosis; PV: polycythemia vera; LS *T*s: lumbar spine *T*-score; LS *Z*s: lumbar spine *Z*-score; FN *T*s: femoral neck *T*-score: FN *Z*s: femoral neck *Z*-score; TH *T*s: total hip *T*-score; TH *Z*s: total hip *Z*-score; n.a.: not assessed due to previous arthrodesis.

## Data Availability

Clinical data are collected in Osteoregistry, which is a property of IRCCS Istituto Ortopedico Galeazzi.

## References

[1] Rossini M., Adami S., Bertoldo F. (2016). Guidelines for the diagnosis, prevention and management of osteoporosis. *Reumatismo*.

[2] Camacho P. M., Petak S. M., Binkley N. (2016). American Association of Clinical Endocrinologists and American College of Endocrinology clinical practice guidelines for the diagnosis and treatment of postmenopausal osteoporosis — 2016. *Endocrine Practice*.

[3] Kanis J. A., Cooper C., Rizzoli R., Reginster J. Y., on behalf of the Scientific Advisory Board of the European Society for Clinical and Economic Aspects of Osteoporosis (ESCEO) and the Committees of Scientific Advisors and National Societies of the International Osteoporosis Foundation (IOF) (2019). European guidance for the diagnosis and management of osteoporosis in postmenopausal women. *Osteoporosis International*.

[4] Pardanani A. (2018). Systemic mastocytosis in adults: 2019 update on diagnosis, risk stratification and management. *American Journal of Hematology*.

[5] Valent P., Akin C., Metcalfe D. D. (2017). Mastocytosis: 2016 updated WHO classification and novel emerging treatment concepts. *Blood*.

[6] Cohen S. S., Skovbo S., Vestergaard H. (2014). Epidemiology of systemic mastocytosis in Denmark. *British Journal of Haematology*.

[7] Merante S., Ferretti V. V., Elena C. (2018). The Italian Mastocytosis Registry: 6-year experience from a hospital-based registry. *Future Oncology*.

[8] Horny H. P., Metcalfe D. D., Akin C., Swerdlow S. H. (2017). Mastocytosis. *WHO classification of tumors of hematopoietic and lymphoid tissues*.

[9] Arber D. A., Orazi A., Hasserjian R. (2016). The 2016 revision to the World Health Organization classification of myeloid neoplasms and acute leukemia. *Blood*.

[10] Pieri L., Bonadonna P., Elena C. (2016). Clinical presentation and management practice of systemic mastocytosis. A survey on 460 Italian patients. *American Journal of Hematology*.

[11] Rossini M., Zanotti R., Bonadonna P. (2011). Bone mineral density, bone turnover markers and fractures in patients with indolent systemic mastocytosis. *Bone*.

[12] Rossini M., Zanotti R., Viapiana O. (2014). Bone involvement and osteoporosis in mastocytosis. *Immunology and Allergy Clinics of North America*.

[13] Veer E., Goot W., Monchy J. G. R., Kluin-Nelemans H. C., Doormaal J. J. (2012). High prevalence of fractures and osteoporosis in patients with indolent systemic mastocytosis. *Allergy*.

[14] Kanis J. A. (1994). Assessment of fracture risk and its application to screening for postmenopausal osteoporosis: synopsis of a WHO report. WHO Study Group. *Osteoporosis International*.

[15] Rossini M., Zanotti R., Orsolini G. (2016). Prevalence, pathogenesis, and treatment options for mastocytosis-related osteoporosis. *Osteoporosis International*.

[16] Schwartz L. B., Bradford T. R., Rouse C. (1994). Development of a new, more sensitive immunoassay for human tryptase: use in systemic anaphylaxis. *Journal of Clinical Immunology*.

[17] Maric I., Sun X. (2019). Advances in diagnosis of mastocytosis and hypereosinophilic syndrome. *Seminars in Hematology*.

[18] Alpay Kanıtez N., Erer B., Doğan Ö. (2015). Osteoporosis and osteopathy markers in patients with mastocytosis. *Turkish Journal of Haematology*.

[19] Acosta-Mérida Á., Ojeda-Bruno S. (2019). Multiple vertebral fractures as the first manifestation of systemic mastocytosis. *Osteoporosis International*.

[20] Delling R. H., Werner M. (2001). Histological characteristics and prevalence of secondary osteoporosis in systemic mastocytosis. A retrospective analysis of 158 cases. *Pathologe*.

[21] Spinnato P., Bazzocchi A., Facchini G. (2018). Vertebral fractures of unknown origin: role of computed tomography-guided biopsy. *International Journal of Spine Surgery*.

[22] Lee A. Y. S. (2020). Elevated serum tryptase in non-anaphylaxis cases: a concise review. *International Archives of Allergy and Immunology*.

[23] Lyons J. J. (2018). Hereditary alpha tryptasemia: genotyping and associated clinical features. *Immunology and Allergy Clinics of North America*.

[24] Greiner G., Sprinzl B., Górska A. (2020). Hereditary alpha tryptasemia is a valid genetic biomarker for severe mediator-related symptoms in mastocytosis. *Blood*.

[25] Lyons J. J., Chovanec J., O’Connell M. P. (2020). Heritable risk for severe anaphylaxis associated with increased *α*-tryptase–encoding germline copy number at TPSAB1. *Journal of Allergy and Clinical Immunology*.

[26] Broesby-Olsen S., Farkas D. K., Vestergaard H. (2016). Risk of solid cancer, cardiovascular disease, anaphylaxis, osteoporosis and fractures in patients with systemic mastocytosis: a nationwide population-based study. *American Journal of Hematology*.

[27] Degboé Y., Eischen M., Nigon D. (2017). Prevalence and risk factors for fragility fracture in systemic mastocytosis. *Bone*.

[28] Degboé Y., Eischen M., Apoil P. A. (2019). Higher prevalence of vertebral fractures in systemic mastocytosis, but not in cutaneous mastocytosis and idiopathic mast cell activation syndrome. *Osteoporosis International*.

[29] Greene L. W., Asadipooya K., Corradi P. F., Akin C. (2016). Endocrine manifestations of systemic mastocytosis in bone. *Reviews in Endocrine & Metabolic Disorders*.

